# Novel Risks of Unfavorable Corticosteroid Response in Patients with Mild-to-Moderate COVID-19 Identified Using Artificial Intelligence-Assisted Analysis of Chest Radiographs

**DOI:** 10.3390/jcm12185852

**Published:** 2023-09-08

**Authors:** Min Hyung Kim, Hyun Joo Shin, Jaewoong Kim, Sunhee Jo, Eun-Kyung Kim, Yoon Soo Park, Taeyoung Kyong

**Affiliations:** 1 Department of Internal Medicine, Division of Infectious Disease, Yongin Severance Hospital, Yonsei University College of Medicine, Yongin-si 16995, Republic of Korea; mhkim16@yuhs.ac (M.H.K.); ysparkok2@yuhs.ac (Y.S.P.); 2 Department of Radiology, Research Institute of Radiological Science and Center for Clinical Imaging Data Science, Yongin Severance Hospital, Yonsei University College of Medicine, Yongin-si 16995, Republic of Korea; lamer-22@yuhs.ac (H.J.S.); ekkim@yuhs.ac (E.-K.K.); 3 Center for Digital Health, Yongin Severance Hospital, Yonsei University College of Medicine, Yongin-si 16995, Republic of Korea; 4 Department of Hospital Medicine, Yongin Severance Hospital, Yonsei University College of Medicine, Yongin-si 16995, Republic of Korea; martins00@yuhs.ac (J.K.); xxshjo@yuhs.ac (S.J.); 5 Department of Biomedical Systems Informatics, Yonsei University College of Medicine, Seoul 03722, Republic of Korea

**Keywords:** artificial intelligence, chest radiograph, corticosteroid responsiveness, COVID-19

## Abstract

The prediction of corticosteroid responses in coronavirus disease 2019 (COVID-19) patients is crucial in clinical practice, and exploring the role of artificial intelligence (AI)-assisted analysis of chest radiographs (CXR) is warranted. This retrospective case–control study involving mild-to-moderate COVID-19 patients treated with corticosteroids was conducted from 4 September 2021, to 30 August 2022. The primary endpoint of the study was corticosteroid responsiveness, defined as the advancement of two or more of the eight-categories-ordinal scale. Serial abnormality scores for consolidation and pleural effusion on CXR were obtained using a commercial AI-based software based on days from the onset of symptoms. Amongst the 258 participants included in the analysis, 147 (57%) were male. Multivariable logistic regression analysis revealed that high pleural effusion score at 6–9 days from onset of symptoms (adjusted odds ratio of (aOR): 1.022, 95% confidence interval (CI): 1.003–1.042, *p* = 0.020) and consolidation scores up to 9 days from onset of symptoms (0–2 days: aOR: 1.025, 95% CI: 1.006–1.045, *p* = 0.010; 3–5 days: aOR: 1.03 95% CI: 1.011–1.051, *p* = 0.002; 6–9 days: aOR; 1.052, 95% CI: 1.015–1.089, *p* = 0.005) were associated with an unfavorable corticosteroid response. AI-generated scores could help intervene in the use of corticosteroids in COVID-19 patients who would not benefit from them.

## 1. Introduction

Severe acute respiratory syndrome coronavirus-2 (SARS-CoV-2) causes COVID-19, a pandemic that has affected the lives of 766 million individuals worldwide [1]. Efforts have been made to mitigate the detrimental effect of this disease, and corticosteroids, a type of immune modulator, have played a pivotal role in reducing mortality rates, as demonstrated in large-scale randomized controlled trials [2,3,4,5]. The mechanism involved in steroid responsiveness lies in its ability to reduce hyperimmune activation triggered by SARS-CoV-2 [6,7]. However, determining and predicting the treatment response to corticosteroids is complicated, making it challenging to identify individuals who will benefit the most from this therapy. These difficulties led to the establishment of criteria for escalating immunomodulator therapy based solely on clinical observation of hypoxia exacerbation [8]. To avoid cases refractory to corticosteroids or rebound phenomena during steroid reduction or after discontinuation, additional methods for predicting corticosteroid responsiveness are required [2,9,10].

The pathophysiologic mechanism of tissue tropism of SARS-CoV-2 through angiotensin-converting enzyme 2 receptor, which damages alveolar epithelial and capillary endothelial cells by an immune reaction, suggests that imaging modality could be used to predict the prognosis of COVID-19 patients [11,12,13]. A study by Liang et al. highlighted the utility of a scoring system that includes a chest radiograph (CXR) as a factor to predict the prognosis of COVID-19 patients [14], while D’Cruz et al. presented opposing views regarding its role [15]. The discrepant results might stem from the absence of standardized measurements of CXR findings that are precise and can be quantified.

The shortcomings of imaging modalities are expected to be averted with the help of deep learning algorithms applied to chest imaging. The role of artificial intelligence (AI)-assisted algorithms in diagnosing and predicting the prognosis of COVID-19 has been widely tested and validated recently [16,17,18,19]. Further usage of this technology in identifying COVID-19 patients with unfavorable corticosteroid response by monitoring AI-based changes in CXR findings is anticipated and deserves further investigation.

Our institution introduced an AI-assisted CXR imaging technology tested and validated in other studies [20,21,22]. This software helps to detect various lesions and provides an abnormality score for each CXR a patient had taken. We aimed to navigate the utility of an AI-generated CXR abnormality (AI-CXR) score in predicting the outcome of patients hospitalized for COVID-19 and treated with corticosteroids.

## 2. Materials and Methods

### 2.1. Study Design and Population

This retrospective case–control study was conducted in a university-affiliated, 500-bed hospital in South Korea. We enrolled mild-to-moderate COVID-19 patients treated with corticosteroids from 4 September 2021, to 30 August 2022. This institution was designated to provide care for mild-to-moderate COVID-19 patients who need hospitalization. Patients whose condition deteriorated and required mechanical ventilation were transferred to other hospitals dedicated to taking care of critically ill patients. Hospitalized patients were treated according to the National Institutes of Health’s COVID-19 Treatment Guidelines [8], except in the early phase of the pandemic when proper treatment guidelines had not been established. Corticosteroids were the most commonly prescribed drugs in the early phase of the pandemic due to easy accessibility in healthcare settings. Enrolled patients were followed up until discharge, and the last follow-up date of the last patient was 6 October 2022. Patients were enrolled according to the following criteria: (1) hospitalized with acute COVID-19 infection confirmed using real-time polymerase chain reaction tests and (2) a history of corticosteroid use of an equivalent dose of dexamethasone 6 mg or less during the SARS-CoV-2 infection regardless of type or date of initiation.

Any patient meeting the following conditions was excluded from the study: (1) under the age of 19 years; (2) without CXR results; and (3) corticosteroid use exceeding an equivalent dose of dexamethasone 6 mg.

The primary endpoint of corticosteroid unresponsiveness was defined as a deterioration of the patient’s condition manifested by the advancement of two or more in the World Health Organization eight-categories-ordinal scale (Table S1) at the time of discharge, or no improvement of a condition if the patient was initially categorized in the 5th category or worse at the time of COVID-19 confirmation.

### 2.2. Data Collection

The data of participants were collected retrospectively by reviewing electronic medical records. Age, sex, underlying condition (diabetes mellitus (DM), chronic obstructive pulmonary disease (COPD), history of myocardial infarction, chronic heart failure, peripheral vascular disease, chronic kidney disease (CKD), chronic liver disease, malignancy of solid organs, leukemia, lymphoma, cerebral vascular disease, dementia, connective tissue disease, peptic ulcer disease, hemiplegia, or human immunodeficiency virus infection), Charlson comorbidity index (CCI), and immunocompromised status determined by Centers for Disease Control and Prevention criteria [23] were recorded. Treatment, history of vaccination, types, and duration of antiviral agents, antibacterial agents, and corticosteroids were reviewed. Laboratory values such as white blood cell count (WBC (10^3^/μL), platelet count (10^3^/μL), lymphocyte percentage (%), C-reactive protein (CRP, mg/L), D-dimer (mcgFEU/mL), Interleukin (IL)-6 (pg/mL), albumin (g/dL), and procalcitonin (PCT, ng/mL) were collected. CXR results were procured as described under the next subheading. CXR and laboratory results were chosen based on the date of onset of symptoms to assess the response according to the course of the disease. Serial results were obtained according to the following categories: (1) 0: 0–2 days from the event; (2) 1: 3–5 days from the event; (3) 2: 6–9 days from the event; and (4) 3: more than 10 days from the event. A single result in each category was included in the analysis.

### 2.3. AI-Based CXR Results

All CXRs were obtained in anteroposterior projection in each patient’s room, as mandated by hospital policy for patients with highly contagious diseases. A commercially available AI-based lesion detection software (Lunit INSIGHT CXR, version 3, Lunit Inc., Seoul, Republic of Korea) was used to obtain the AI-CXR score of lung lesions. This software used certified convoluted neural network architecture in its development and is capable of detecting a total of eight lesions on CXRs, including pulmonary nodule, consolidation, pneumothorax, fibrosis, atelectasis, cardiomegaly, pleural effusion, and pneumoperitoneum [24,25]. Since consolidation and pleural effusion were known to be associated with COVID-19 pneumonia, we extracted consolidation and pleural effusion AI-CXR scores from the AI server, which were integrated into all CXRs taken throughout hospitalization. The abnormality score by the AI software is presented as a percentage ranging from 0 to 100%, which indicates the AI-decided probability of CXR having the lesion. Our hospital used a cutoff value of 15% for the abnormality score to decide the presence of the lesion according to vendors and another study [26]. Using this cutoff value, this software determines that the lesion is present on the CXR and displays a contour map along with the abnormality score as described in Figure S1.

### 2.4. Statistical Analysis

Participants with favorable and unfavorable corticosteroid responsiveness were compared. Baseline characteristics were compared using Mann–Whitney U test, independent samples *t*-test for continuous variables, and χ^2^ test or Fisher’s exact test for categorical variables. Continuous variables are expressed as means ± standard deviation, or medians (interquartile ranges) and categorical variables as numbers with percentages for the description of baseline characteristics. A generalized estimating equation model with logit links was used to analyze whether repeated-measured CXR results and laboratory data influenced the primary outcome. Univariate and multivariate logistic regression tests were performed to determine the change in the performance of the fitted model for each time category. Covariates for the multivariable logistic model were chosen based on *p*-value < 0.05 in a univariate analysis and clinical significance. Additionally, subgroup analysis involving patients with hypoxia and categorized according to the date of COVID-19 confirmation was conducted using a model that included AI-CXR score as a predictor. The association of the AI-CXR score with other biomarkers was estimated using linear regression analysis. A *p*-value < 0.05 was considered statistically significant. Cases with missing values in any category were excluded from the analysis. The prediction accuracy of the AI-CXR score was assessed using the area under the receiver operating characteristic (ROC) curve. For the statistical analysis, we used R (version 4.2.2, Foundation for Statistical Computing, Vienna, Austria) and SPSS (version 26.0, IBM Corp., Armonk, NY, USA).

## 3. Results

### 3.1. Baseline Characteristics

**Figure 1:**
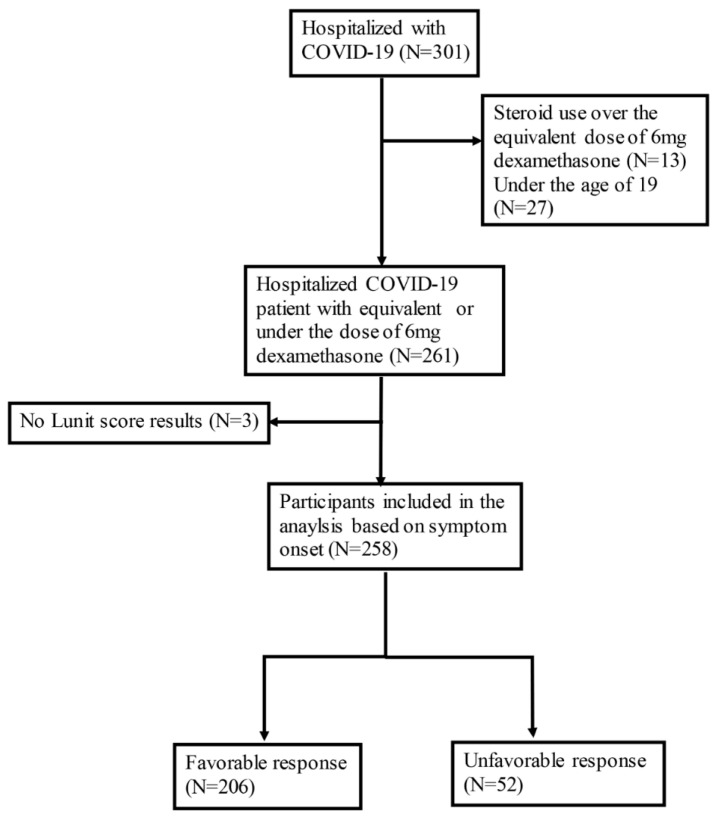
Flow chart of patient enrollment in the analysis.

Of the 752 COVID-19 patients hospitalized during the study period, 274 received corticosteroid treatment. Among them, 13 who exceeded the dose of dexamethasone 6 mg were excluded. Additionally, three patients were excluded due to lack of CXR results. As a result, 258 participants were included in the analysis with 52 being classified as having an unfavorable response and 206 as having a favorable response to corticosteroid therapy (Figure 1). The average age of participants was 64.21 ± 18.88 years, with 147 (57.0%) being male. Among those who were enrolled, 76 (29.5%) patients had DM, 7 (2.7%) had CKD, and 42 (16.3%) had a malignancy. As for the patient allocation, 51 (19.8%) were transferred due to deteriorating conditions and 1 (0.4%) patient died. Patients with unfavorable corticosteroid response were older (69.67 ± 16.52 vs. 62.83 ± 19.19, *p* < 0.01), had higher CCI values (1.5 [0–4] vs. 1 [0–2], *p* < 0.01), and had a greater proportion of those with immunocompromised status (17 (32.7%) vs. 27 (13.1%), *p* < 0.01) than patients with favorable corticosteroid response. The vaccination rate did not differ between the two groups (21 (43.8%) vs. 97 (50.3%), *p* = 0.11); however, a higher proportion of patients with unfavorable responses received antiviral (40 (76.9%) vs. 112 (54.4%), *p* < 0.01) and antibacterial treatments (50 (96.2%) vs. 151 (73.3%), *p* < 0.01). Most patients were treated with dexamethasone (243/258, 94.2%), with three participants (3/206, 1.5%) in the favorable response group receiving less than the equivalent dose of dexamethasone 6 mg (Table 1).

### 3.2. AI-CXR Score as a Factor Associated with Unfavorable Corticosteroid Response

**Figure 2:**
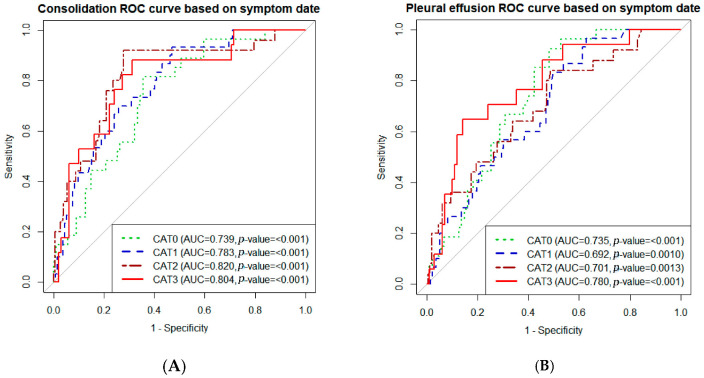
Receiver operating characteristic (ROC) curve of artificial intelligence-generated chest radiograph (AI-CXR) scores for predicting corticosteroid responsiveness. (**A**) ROC curve of the consolidation score. (**B**) ROC curve of the pleural effusion score. ROC curves of sequential AI-CXR score were drawn according to time category. Abbreviations: CAT 0: category 0, 0–2 days from the onset of symptoms; CAT 1: category 1, 3–5 days from the onset of symptoms; CAT 2: category 3, 6–9 days from the onset of symptoms; CAT 3, more than 10 days from the onset of symptoms; AUC, area under the curve.

The pleural effusion score in category 2 (adjusted odds ratio (aOR) 1.022, 95% confidence interval (CI) 1.003–1.042, *p* = 0.02) and consolidation score in category 0–2 (category 0: aOR 1.025, 95% CI 1.006–1.045, *p* = 0.01; category 1: aOR 1.03 95% CI 1.011–1.051, *p* < 0.01; category 2: aOR 1.052, 95% CI 1.015–1.089, *p* < 0.01) were associated with an unfavorable outcome (Table 2). A box plot of the AI-CXR score according to the endpoint and time category is shown in Figure S2. The prediction accuracy of the AI-CXR score was estimated using ROC curve analysis. The area under the curve for consolidation score ranged from 0.739 to 0.855 and that of pleural effusion ranged from 0.692 to 0.809 and, hence, has a significant power to predict the outcome of unfavorable corticosteroid response (Figure 2).

The results of the subgroup analysis involving patients with conditions of concern are presented in Figures S3 and S4. Consolidation scores remained relevant in predicting corticosteroid responsiveness in patients with hypoxia and patients diagnosed in the Delta variant-dominant period. Pleural effusion score was associated with the outcome in the Omicron variant-dominant period.

### 3.3. Association between AI-CXR Scores and Other Laboratory Tests Correlated with Unfavorable Corticosteroid Response

High CRP level was associated with unfavorable corticosteroid response across all time categories. Low lymphocyte percentages also differed between unfavorable and favorable corticosteroid response groups, but only in category 2 (aOR 0.914, 95% CI 0.851–0.982, *p* = 0.01) and category 3 (aOR 0.857, 95% CI 0.752–0.970, *p* = 0.02) in grouping (Table S2). The differences in values between the two groups are presented in Table S3.

Regarding variables that had a linear correlation with the AI-CXR score, CRP, albumin, and lymphocyte percentage showed a close correlation across all time categories with both consolidation and pleural effusion scores. The extent of correlation is expressed as a parameter estimate (Table 3).

## 4. Discussion

The findings of this study suggested that AI-based software applied to CXR could predict treatment response by utilizing previously underrecognized factors. The abnormality scores of pleural effusion and consolidation, generated by a commercially available AI software, demonstrated good predictive performance for corticosteroid responsiveness in COVID-19 patients. The positive correlation between AI-CXR scores and other biomarkers associated with an unfavorable response suggested the reliability of this technology.

To select patients who would benefit the most from corticosteroid treatment, risk factors associated with adverse outcomes need to be investigated. Previous studies have focused on laboratory results and underlying conditions instead of imaging findings. Murakami et al. proposed severe respiratory failure and high-soluble IL-2 receptor, lactate dehydrogenase, and CRP levels as factors associated with adverse outcomes [27]. A study using deep learning algorithms in predicting corticosteroid responsiveness also included laboratory results such as lymphocyte percentage, PCT, and tumor necrosis factor α, IL-1β, IL-2 receptor, IL-6, IL-8, IL-10, and CRP levels [28]. Our study is different from these studies in that we attempted to use CXR imaging as the main tool for outcome prediction. Considering the pathogenesis of COVID-19 and the mechanism of action of corticosteroids, the use of an imaging modality is the better option for assessing corticosteroid responsiveness. Our study shows that quantified scores presented by AI systems could be used to predict corticosteroid responsiveness.

The efficacy of corticosteroids is dependent on their ability to reduce cytokines by suppressing inflammatory cells involved in adaptive immunity. This would prevent alveolar damage triggered by the reaction, which is likely to be detected by the imaging modality [29,30,31,32,33,34]. Recent studies involving AI-assisted image analysis shed light on the utilization of the technique in diagnosing and predicting the prognosis of COVID-19 by quantifying opacification of the alveolar system or interstitial tissue and precise localization with augmentation [19,35]. These characteristics enable the implementation of a simple imaging modality such as CXR in a setting where a complex imaging technique is unavailable due to the high risk of disease exposure. In this study, we showed that quantified scores indicating the probability, based on a simple modality as CXR, can be used to identify patients with poor responses. Since consolidation is a frequently observed finding in COVID-19-associated pneumonia, typically appearing around 6–7 days after the onset of symptoms, it is understandable that there is an association between the consolidation score within 10 days after the onset of symptoms and the treatment outcome [36]. This is also consistent with the recent report of an association of between the extent of pneumonia in CXR and poor outcomes [13]. Notably, the gap in pleural effusion score widened approximately a week after symptom onset, when viral replication usually phases out with hyper-immune activation phasing in. Pleural effusion is not directly associated in COVID-19; however, it could be associated with hyper-immune activation such as multi-system inflammatory syndrome through endothelial damage [37,38]. Our study presents tangible evidence of the proposed mechanism of corticosteroid action. Therefore, we can interpret our finding as an aberrant immune reaction expressed as consolidation at an early stage that led to pleural effusion at a later stage that could not be slowed down through corticosteroid administration, resulting in poor treatment response. Considering that disease progression is associated with hyperimmune activation, patients with consolidation at approximately one week from the onset of symptoms and unimproved pleural effusion should be considered for preliminary therapy with other immune-modulating agents.

Our results indicate that AI-CXR scores linearly correlated with other biomarkers associated with unfavorable outcomes. This would help resolve questions related to AI and its implementation in clinical practice. Consistent with previously identified prognostic factors of COVID-19 [27,39,40], CRP level and lymphocyte percentage were associated with unfavorable treatment responses in this analysis. AI-CXR scores had a linear correlation with high CRP, low lymphocyte percentages, and low albumin levels. The association between high AI-CXR scores to these variables suggests that AI-CXR scores can be used to describe disease severity.

AI-CXR scores showed a similar significance in subgroup analysis including only patients who required oxygen therapy, except for pleural effusions score in category 2. This might be attributed to the small sample size. Further studies with a larger number of participants are required to verify whether the AI-CXR score can be reliably applied to those who need corticosteroid treatment as the current guidelines stipulate. AI-CXR scores had a significant effect on the outcome in both the Delta and Omicron variant-dominant periods. In South Korea, the Omicron variant gained dominance by the first week of January 2022. It is noteworthy that different CXR findings were significant in terms of corticosteroid responsiveness during each period. In the Delta variant-dominant period, consolidation scores were associated with unfavorable corticosteroid response, while pleural effusion scores predicted it in the Omicron variant-dominant period. The Omicron variant is known for atypical presentation on chest CT compared to the Delta variant, despite its reduced virulence [41]. This might indicate that the Omicron strain could still be a threat to patients with weakened immune systems by replication of similar pathophysiologic damage to a patient’s respiratory system. Application of AI-CXR scores to other pathogenic organisms is expected.

This study has some limitations. First, most of the patients in the unfavorable group had been transferred due to critical conditions; therefore, their outcomes are not known, and thus, the results of the unfavorable group may have been overestimated. Second, because of the retrospective design and small sample size, missing values in laboratory and CXR tests could have affected the statistical power of this study. However, we believe our results are important because we obtained statistical significance when we excluded the missing data due to early transfer or discharge from the analysis instead of using data imputation. Third, the diagnostic accuracy of AI-based software was not evaluated according to the radiologists’ reports or CT scans because this was out of the scope of our study. However, this software is already well-known for its high diagnostic performance in other studies [42,43] and we attempted to investigate the robustness of the AI-CXR score by examining the correlation with other biomarkers instead. Fourth, we used scores showing the possibility of the presence of the lesions presented by AI for the analysis. It is debatable whether abnormality scores presented by AI can constitute an absolute quantitative value to represent disease extent or severity, unlike other quantitative imaging markers, due to the undefinable characteristics of AI itself. However, we assumed that increased area or opacity on CXR could increase the possibility of prediction by AI and decided to set this value for monitoring treatment response throughout this study.

## 5. Conclusions

This study demonstrated a negative correlation between corticosteroid response and AI-generated pleural effusion scores obtained approximately a week later, as well as consolidation scores during the early stage of the onset of symptoms. Patients with signs of poor response should be considered for pre-treatment with other immune-modulating agents. Further validation of the technology involving patients with different disease entities is warranted.

## Figures and Tables

**Table 1:** Baseline characteristics of the hospitalized COVID-19 patients treated with corticosteroids.

	Total	Unfavorable * (*n* = 52)	Favorable (*n* = 206)	*p*-Value
Age (years)	64.21 ± 18.88	69.67 ± 16.52	62.83 ± 19.19	<0.001
Sex (male), *n* (%)	147 (57.0)	42 (80.8)	105 (51.0)	<0.001
Re-infection, *n* (%)	2 (0.8)	0 (0.0)	2 (1.0)	0.371 ^†^
Comorbidities, *n* (%)				
DM	76 (29.5)	17 (32.7)	59 (28.6)	0.220
COPD	19 (7.4)	4 (7.7)	15 (7.3)	0.882 ^†^
CHF	16 (6.2)	3 (5.8)	13 (6.3)	0.873 ^†^
CKD	7 (2.7)	2 (3.8)	5 (2.4)	0.243 ^†^
Chronic liver Dz.	5 (1.9)	1 (1.9)	4 (1.9)	1.00 ^†^
Malignancy	42 (16.3)	14 (26.9)	28 (13.6)	<0.001
CCI	1 [0–3]	1.5 [0–4]	1 [0–2]	<0.001
Immunocompromised, *n* (%)	44 (17.1)	17 (32.7)	27 (13.1)	<0.001
Outcomes				
Condition at discharge, *n* (%)				<0.001
Normal discharge	205 (79.4)	0 (0.0)	205 (99.5)	
Transfer	51 (19.8)	51 (98.1)	0 (0.0)	
Death	1 (0.4)	1 (1.9)	0 (0.0)	
Others	1 (0.4)	0 (0.0)	1 (0.5)	
Hospital days	8 [6–12]	4 [1–11.75]	8 [6–12]	<0.001
Treatments				
Oxygen requirements, *n* (%)				<0.001
None	99 (38.4)	0 (0.0)	99 (48.1)	
Low-flow oxygen	101 (39.1)	3 (5.8)	98 (47.6)	
High-flow oxygen	55 (21.3)	46 (88.5)	9 (4.4)	
Mechanical ventilation	3 (1.2)	3 (5.8)	0 (0.0)	
Monoclonal antibody, *n* (%)	10 (3.9)	0 (0.0)	10 (4.9)	<0.001
Tocilizumab, *n* (%)	0 (0.0)	0 (0.0)	0 (0.0)	
Antiviral agents, *n* (%)	152 (58.9)	40 (76.9)	112 (54.4)	<0.001
Remdesivir	146 (96.1)	39 (97.5)	107 (95.5)	
Nirmatrevir/lopinavir	3 (2.0)	1 (2.5)	2 (1.8)	
Molnuprevir	3 (2.0)	0 (0.0)	3 (2.7)	
Antibacterial agents, *n* (%)	201 (77.9)	50 (96.2)	151 (73.3)	<0.001
Vaccination, *n* (%)	118 (49.0)	21(43.8)	97 (50.3)	0.115
Primary vaccination	97 (82.2)	20 (95.2)	77 (79.4)	
Booster	21 (17.8)	1(4.8)	20 (20.6)	
Corticosteroid Treatment				
Types, *n* (%)				1.000
Dexamethasone	243 (94.2)	51 (98.1)	192 (93.2)	
Methylprednisolone	8 (3.1)	1 (1.9)	7 (3.4)	
Prednisolone	4 (1.5)	0 (0.0)	4 (1.9)	
Hydrocortisone	3 (1.1)	0 (0.0)	3 (1.4)	
Doses, *n* (%)				0.300
6 mg equivalent	255 (98.5)	52 (100.0)	203 (98.5)	
less	3 (1.2)	0 (0.0)	3 (1.5)	
Days of steroid initiation ^‡^	4 [2–7]	3 [2–6]	4 [2–7]	0.271
Treatment duration	5 [4–8]	3 [1.25–8]	6 [4–8]	0.350

Data are expressed as mean ± standard deviation, median [Q1–Q3], or number with percentages. Abbreviations: DM, diabetes mellitus; COPD, chronic obstructive pulmonary disease; CHF, chronic heart failure; CKD, chronic kidney disease; Dz., disease; CCI, Charlson comorbidity index. * Unfavorable corticosteroid responsiveness was defined as either advancement of two or more of the eight-categories-ordinal scale established by the World Health Organization or no improvement from the initial 5th or worse category. ^†^ *p*-value was calculated using Fisher’s exact test; ^‡^ Days between steroid initiation and COVID-19 confirmation.

**Table 2:** Association of artificial intelligence-generated chest radiograph abnormality score with unfavorable corticosteroid response according to time category.

	Variables	Univariate		Multivariable	
		OR * (95% CI)	*p*-Value	aOR ^†^ (95% CI)	*p*-Value
Total	Consolidation score (%)	1.030 (1.017–1.042)	<0.001	1.022 (1.010–1.035)	<0.001
	Pleural effusion score (%)	1.020 (1.009–1.032)	0.001	1.013 (1.001–1.026)	0.040
Category 0 ^‡^	Consolidation score (%)	1.025 (1.011–1.039)	<0.001	1.025 (1.006–1.045)	0.010
	Pleural effusion score (%)	1.016 (0.999–1.033)	0.068	1.003 (0.984–1.021)	0.780
Category 1 ^§^	Consolidation score (%)	1.035 (1.018–1.053)	<0.001	1.03 (1.011–1.051)	0.002
	Pleural effusion score (%)	1.020 (1.004–1.035)	0.013	1.017 (0.999–1.035)	0.070
Category 2 ^‖^	Consolidation score (%)	1.057 (1.022–1.093)	0.001	1.052 (1.015–1.089)	0.005
	Pleural effusion score (%)	1.025 (1.010–1.040)	0.001	1.022 (1.003–1.042)	0.020
Category 3 ^¶^	Consolidation score (%)	1.058 (1.006–1.113)	0.028	1.033 (0.988–1.080)	0.158
	Pleural effusion score (%)	1.022 (1.006–1.039)	0.006	1.003 (0.979–1.027)	0.809

Values with statistical significance of *p* < 0.05 were presented with bold type. Abbreviations: OR, odds ratio; aOR, adjusted odds ratio; CI, confidence interval. * OR was calculated using a generalized estimating equation for all measurements involved or logistic regression analysis in categorical measurements. ^†^ aOR was adjusted for age, sex, Charlson comorbidity index, immune status, vaccination status, antiviral agent usage, and antibacterial agent usage. ^‡^ 0–2 days from the onset of symptoms. ^§^ 3–5 days from the onset of symptoms. ^‖^ 6–9 days from the onset of symptoms. ^¶^ More than 10 days from the onset of symptoms.

**Table 3:** The relationship of artificial intelligence-generated chest radiograph abnormality score with other laboratory variables.

	Variables	Consolidation Score			Pleural Effusion Score		
		Parameter Estimate	t	*p*-Value	Parameter Estimate	t	*p*-Value
Category 0 *	WBC (10^3^/μL)	1.27	1.70	0.09	0.803	1.69	0.09
	PLT (10^3^/μL)	0.01	0.22	0.82	0.07	3.01	<0.01
	Lymphocyte (%)	−0.23	−0.61	0.54	−0.12	−0.49	0.63
	CRP (mg/L)	0.15	2.5	0.01	0.10	2.66	<0.01
	Albumin (g/dL)	−27.77	−5.87	<0.01	−7.05	−2.2	0.03
	IL-6 (pg/mL)	0.72	1.32	0.24	0.00	0	0.99
	D-dimer (mcgFEU/mL)	1.71	1.08	0.28	0.37	0.36	0.72
	Procalcitonin (ng/mL)	2.02	1.88	0.07	1.49	1.99	0.054
Category 1 ^†^	WBC (10^3^/μL)	2.46	2.65	<0.01	3.09	6.57	<0.01
	PLT (10^3^/μL)	−0.01	−0.17	0.87	0.06	3.00	<0.01
	Lymphocyte (%)	−1.12	−4	<0.01	−0.46	−2.92	<0.01
	CRP (mg/L)	0.21	4.75	<0.01	0.08	3.09	<0.01
	Albumin (g/dL)	−24.59	−4.74	<0.01	−13.41	−4.69	<0.01
	IL-6 (pg/mL)	0.01	0.21	0.83	0.01	5.88	<0.01
	D-dimer (mcgFEU/mL)	6.84	1.41	0.17	4.25	1.38	0.17
	Procalcitonin (ng/mL)	5.12	1.67	0.11	3.82	1.67	0.11
Category 2 ^‡^	WBC (10^3^/μL)	1.42	2.11	0.04	1.02	2.17	0.03
	PLT (10^3^/μL)	−0.001	−0.03	0.97	0.01	0.41	0.68
	Lymphocyte (%)	−1.26	−6.61	<0.01	−0.33	−2.19	0.03
	CRP (mg/L)	0.20	4.57	<0.01	0.08	3.09	0.27
	Albumin (g/dL)	−16.08	−3.33	<0.01	−18.61	−5.83	<0.01
	IL-6 (pg/mL)	0.02	1.34	0.20	−0.01	−0.53	0.60
	D-dimer (mcgFEU/mL)	8.93	1.09	0.29	1.75	0.35	0.73
	Procalcitonin (ng/mL)	0.23	1.03	0.31	−0.11	−0.40	0.69
Category 3 ^§^	WBC (10^3^/μL)	1.16	1.69	0.09	−0.19	−0.38	0.70
	PLT (10^3^/μL)	−0.01	−0.26	0.80	−0.04	−2.76	<0.01
	Lymphocyte (%)	−0.67	−2.49	0.01	−0.31	−1.61	0.11
	CRP (mg/L)	0.08	1.79	0.08	0.07	2.11	0.04
	Albumin (g/dL)	−17.19	−3.41	<0.01	−10.72	−2.80	<0.01
	IL-6 (pg/mL)	0.34	2.80	0.03	−0.14	−0.38	0.72
	D-dimer (mcgFEU/mL)	8.06	0.93	0.36	4.47	0.67	0.68
	Procalcitonin (ng/mL)	−2.86	−0.19	0.85	4.45	0.42	0.68

The extent of association was presented as a parameter estimate, which was calculated using a linear regression. Values with statistical significance of *p* < 0.05 were presented with bold type. Abbreviations: WBC, white blood cell count; PLT, platelet count; CRP, c-reactive protein; IL-6, interleukin six; NA, not applicable. * 0–2 days from the symptom onset. ^†^ 3–5 days from the symptom onset. ^‡^ 6–9 days from the symptom onset. ^§^ More than 10 days from the symptom onset.

## Data Availability

The dataset supporting the conclusions of this article is included within supplemental data (raw data used in the analysis).

## Supplementary Materials

The following supporting information can be downloaded at: https://www.mdpi.com/article/10.3390/jcm12185852/s1, Table S1: Eight-categories-ordinal scale of the World Health Organization; Table S2: Association of laboratory tests with unfavorable corticosteroid response according to time category; Table S3: The differences in laboratory values according to corticosteroid responsiveness; Figure S1: Examples of artificial intelligence-generated abnormality scores incorporated in patients’ chest radiographs; Figure S2: Differences in artificial intelligence-generated chest radiograph scores according to corticosteroid responsiveness and time category; Figure S3: Association between consolidation score and unfavorable corticosteroid responsiveness according to the time category and subgroup; Figure S4: Association between pleural effusion score and unfavorable corticosteroid responsiveness according to the time category and subgroups. Reference [44] is cited in Supplementary Materials.

## Abbreviations

SARS-CoV-2	devere acute respiratory syndrome coronavirus-2
CXR	chest radiographs
AI	artificial intelligence
AI-CXR score	artificial intelligence-generated chest radiograph abnormality score
DM	diabetes mellitus
COPD	chronic obstructive pulmonary disease chronic kidney disease
CKD	chronic kidney disease
CCI	Charlson comorbidity index
WBC	white blood cell count
CRP	C-reactive protein
IL	interleukin
PCT	procalcitonin
ROC	receiver operating characteristic
aOR	adjusted odds ratio
CI	confidence interval
