# Supplementary material for: Novel Risks of Unfavorable Corticosteroid Response in Patients with Mild-to-Moderate COVID-19 Identified Using Artificial Intelligence-Assisted Analysis of Chest Radiographs
Source: J Clin Med. 2023 Sep 8;12(18):5852. doi: 10.3390/jcm12185852 (PMC10532025; doi:10.3390/jcm12185852)
Supplement: Supplementary file 1 [file jcm-12-05852-s001.zip › jcm-2553580-supplementary.pdf]

Supplementary Materials

This Supplementary has been provided by the authors to supplement readers with additional information about this work.

Table S1. Eight-categories-ordinal scale of the World Health Organization [1].

Patient Status	Descriptor	Category
**Ambulatory**	**No limitations on activities**	**1**
	**Limitation of activities**	**2**
**Hospitalized, mild disease**	**Hospitalized, no oxygen therapy**	**3**
	**Oxygen by mask or nasal prongs**	**4**
**Hospitalized, severe disease**	**Non-invasive ventilation or high-flow oxygen**	**5**
	**Intubation and mechanical ventilation**	**6**
	**Additional organ life support such as renal replacement therapy and extracorporeal membrane oxygenation**	**7**
**Dead**	**Death**	**8**

Table S2. Association of laboratory tests with unfavorable corticosteroid response according to time category.

	Variables	Univariate		Multivariable	
		OR* (95% CI)	p-value	aOR† (95% CI)	p-value
Total	WBC (10^3/uL)	1.087 (1.011-1.167)	**<0.001**	1.054 (0.972-1.142)	0.200
	PLT (10^3/uL)	0.997 (0.991-1.002)	0.143		
	Lymphocyte (%)	0.937 (0.898-0.977)	**0.002**	0.962 (0.922-1.003)	0.070
	CRP (mg/L)	1.016 (1.010-1.022)	**<0.001**	1.015 (1.008-1.022)	**<0.001**
	Albumin (g/dL)	0.353 (0.210-0.592)	**<0.001**	0.616 (0.357-1.063)	0.082
	IL-6 (pg/mL)	1.000 (0.999-1.001)	0.775		
	D-dimer (mcgFEU/mL)	1.260 (0.965-1.646)	0.090		
	Procalcitonin (ng/mL)	1.007 (0.973-1.042)	0.692		
Category 0‡	WBC (10^3/uL)	1.057 (0.971-1.151)	0.198		
	PLT (10^3/uL)	0.999 (0.994-1.003)	0.545		
	Lymphocyte (%)	0.970 (0.923-1.020)	0.234		
	CRP (mg/L)	1.013 (1.005-1.020)	**0.001**	1.011 (1.001-1.021)	**0.038**
	Albumin (g/dL)	0.322 (0.160-0.646)	**0.001**	0.342 (0.132-0.886)	**0.027**
	IL-6 (pg/mL)	NA	NA		
	D-dimer (mcgFEU/mL)	1.154 (0.935-1.426)	0.183		
	Procalcitonin (ng/mL)	1.165 (0.993-1.366)	0.061		
Category 1§	WBC (10^3/uL)	1.133 (1.018-1.261)	**0.022**	1.107 (0.973-1.260)	0.132
	PLT (10^3/uL)	1.000 (0.995-1.005)	0.926		

	Lymphocyte (%)	0.959 (0.922-0.998)	**0.038**	0.987 (0.948-1.027)	0.551
	CRP (mg/L)	1.013 (1.00-1.020)	**<0.001**		
	Albumin (g/dL)	0.343 (0.170-0.691)	0.030		
	IL-6 (pg/mL)	0.999 (0.991-1.008)	0.999		
	D-dimer (mcgFEU/mL)	1.748 (0.983-3.109)	0.057		
	Procalcitonin (ng/mL)	1.283 (0.882-1.866)	0.192		
Category 2[‖]	WBC (10^3/uL)	1.046 (0.940-1.164)	0.406		
	PLT (10^3/uL)	0.994 (0.988-0.999)	**0.031**	0.998 (0.991-1.004)	0.433
	Lymphocyte (%)	0.916 (0.864-0.970)	**0.003**	0.914 (0.851-0.982)	**0.014**
	CRP (mg/L)	1.017 (1.009-1.024)	**<0.001**	1.016 (1.007-1.025)	**<0.001**
	Albumin (g/dL)	0.299 (0.128-0.698)	**0.005**	0.361 (0.107-1.219)	0.101
	IL-6 (pg/mL)	1.000 (0.996-1.004)	0.878		
	D-dimer (mcgFEU/mL)	1.187 (0.227-6.204)	0.839		
	Procalcitonin (ng/mL)	0.708 (0.077-6.505)	0.761		
Category 3[¶]	WBC (10^3/uL)	1.026 (1.060-1.372)	**0.005**	1.249 (1.052-1.482)	**0.011**
	PLT (10^3/uL)	0.995 (0.989-1.000)	0.052		
	Lymphocyte (%)	0.82 (0.734-0.916)	**<0.001**	0.857 (0.752-0.970)	**0.020**
	CRP (mg/L)	1.031 (1.013-1.046)	**<0.001**	1.029 (1.009-1.049)	**0.005**
	Albumin (g/dL)	0.154 (0.049-0.477)	**0.001**	0.266 (0.049-1.428)	0.122
	IL-6 (pg/mL)	NA	NA		
	D-dimer (mcgFEU/mL)	3.508 (0.417-29.504)	0.248		

Procalcitonin (ng/mL)	39.177 (0.771-1990.064)	0.067

Values with statistical significance of $p < 0.05$ were presented with bold type. Abbreviations: OR, odds ratio; aOR, adjusted odds ratio; CI, confidence interval; WBC, white blood cell count; PLT, platelet count; CRP, C-reactive protein; IL-6, interleukin six; NA, not applicable.

[*] OR was calculated using a generalized estimating equation for all measurements involved or logistic regression analysis in categorical measurements.

[†] aOR was adjusted for age, sex, charlson comorbidity index, immune status, vaccination status, antiviral agent usage, and antibacterial agent usage.

[‡] 0-2 days from the onset of symptoms.

[§] 3-5 days from the onset of symptoms.

[‖] 6-9 days from the onset of symptoms.

[¶] More than 10 days from the onset of symptoms.

Table S3. The differences in laboratory values according to corticosteroid responsiveness.

	Variables	Based on symptom	
		Unfavorable*	Favorable
Category 0†	WBC (10^3/uL)	6.79 [4.977-10.36]	6.23 [4.55-8.31]
	PLT (10^3/uL)	187.50 [141.25-251.75]	194.00 [155.26-247.25]
	Lymphocyte (%)	15.90 [10.45-19.53]	16.20 [10.80-22.08]
	CRP (mg/L)	71.20 [32.50-128.05]	20.25 [8.00-47.65]
	Albumin (g/dL)	3.54±0.80	4.043±0.60
	IL-6 (pg/mL)	100.05 [85.10-NA]	17.70 [7.36-30.45]
	D-dimer (mcgFEU/mL)	0.63 [0.32-1.95]	0.43 [0.23-0.93]
	Procalcitonin (ng/mL)	1.19 [0.44-10.69]	0.12 [0.05-0.24]
Category 1‡	WBC (10^3/uL)	6.33 [4.84-9.80]	5.31 [3.79-7.44]
	PLT (10^3/uL)	165.00 [101.00-244.00]	173.00 [141.00-216.00]
	Lymphocyte (%)	13.15 [5-21.52]	20.50 [13.25-31.05]
	CRP (mg/L)	72.00 [50.60-139.30]	26.05 [7.70-59.53]
	Albumin (g/dL)	3.37±0.76	3.82±0.59
	IL-6 (pg/mL)	73.4	26.10 [6.81-72.90]
	D-dimer (mcgFEU/mL)	1.07 [0.39-2.73]	0.31 [0.20-0.61]
	Procalcitonin (ng/mL)	0.69 [0.45-3.49]	0.11 [0.05-1.14]
Category 2§	WBC (10^3/uL)	7.33 [3.53-11.04]	6.16 [4.51-8.37]
	PLT (10^3/uL)	153.00 [80.00-218.50]	201.00 [147.00-264.00]
	Lymphocyte (%)	10.300 [7.00-21.50]	19.60 [13.10-26.95]

	CRP (mg/L)	76.65 [47.60-435.52]	16.40 [6.70-45.08]
	Albumin (g/dL)	3.19±0.71	3.58±0.51
	IL-6 (pg/mL)	98.20 [70.40-NA]	24.30 [13.88-71.87]
	D-dimer (mcgFEU/mL)	0.435 [0.35-0.70]	0.41 [0.21-0.52]
	Procalcitonin (ng/mL)	0.24 [0.12-0.38]	0.11 [0.03-0.17]
Category 3[ǀ]	WBC (10^3/uL)	9.54 [7.49-15.14]	7.04 [5.45-9.43]
	PLT (10^3/uL)	99.00 [73.00-261.00]	256 [195.75-319.25]
	Lymphocyte (%)	8.40 [3.90-12.70]	19.00 [12.50-25.93]
	CRP (mg/L)	97.85 [58.1-202.10]	15.90 [4.95-29.95]
	Albumin (g/dL)	2.89±0.72	3.48±0.47
	IL-6 (pg/mL)	NA	33.79 [6.54-65.50]
	D-dimer (mcgFEU/mL)	0.755 [0.362-1.342]	0.40 [0.29-0.70]
	Procalcitonin (ng/mL)	0.13 [0.09-0.75]	0.07 [0.04-0.14]

Data are expressed as mean ± standard deviation or median [Q1-Q3]. Abbreviations: OR, odds ratio; CI, confidence interval; WBC, white blood cell count; PLT, platelet count; CRP, C-reactive protein; IL-6, interleukin six; NA, not applicable.

[*]Unfavorable corticosteroid responsiveness was defined as either advancement of two or more of the eight-categories-ordinal scale established by WHO or no improvement from the initial 5th or worse category.

[†]0-2 days from the onset of symptoms.

[‡]3-5 days from the onset of symptoms.

[§]6-9 days from the onset of symptoms.

[ǀ]more than 10 days from the onset of symptoms.

Figure legends

Figure S1. Examples of artificial intelligence-generated abnormality scores incorporated in patients′ chest radiographs show the probability of the presence of abnormal lesions. (A) An 82-year-old male patient with unfavorable results (consolidation score, left 96%, consolidation score, right 96%, pleural effusion score, left 42%). (B) An 83-year-old female patient with the favorable response (pleural effusion score, left 72%).

Abbreviations: Csn, consolidation; PEf, pleural effusion.

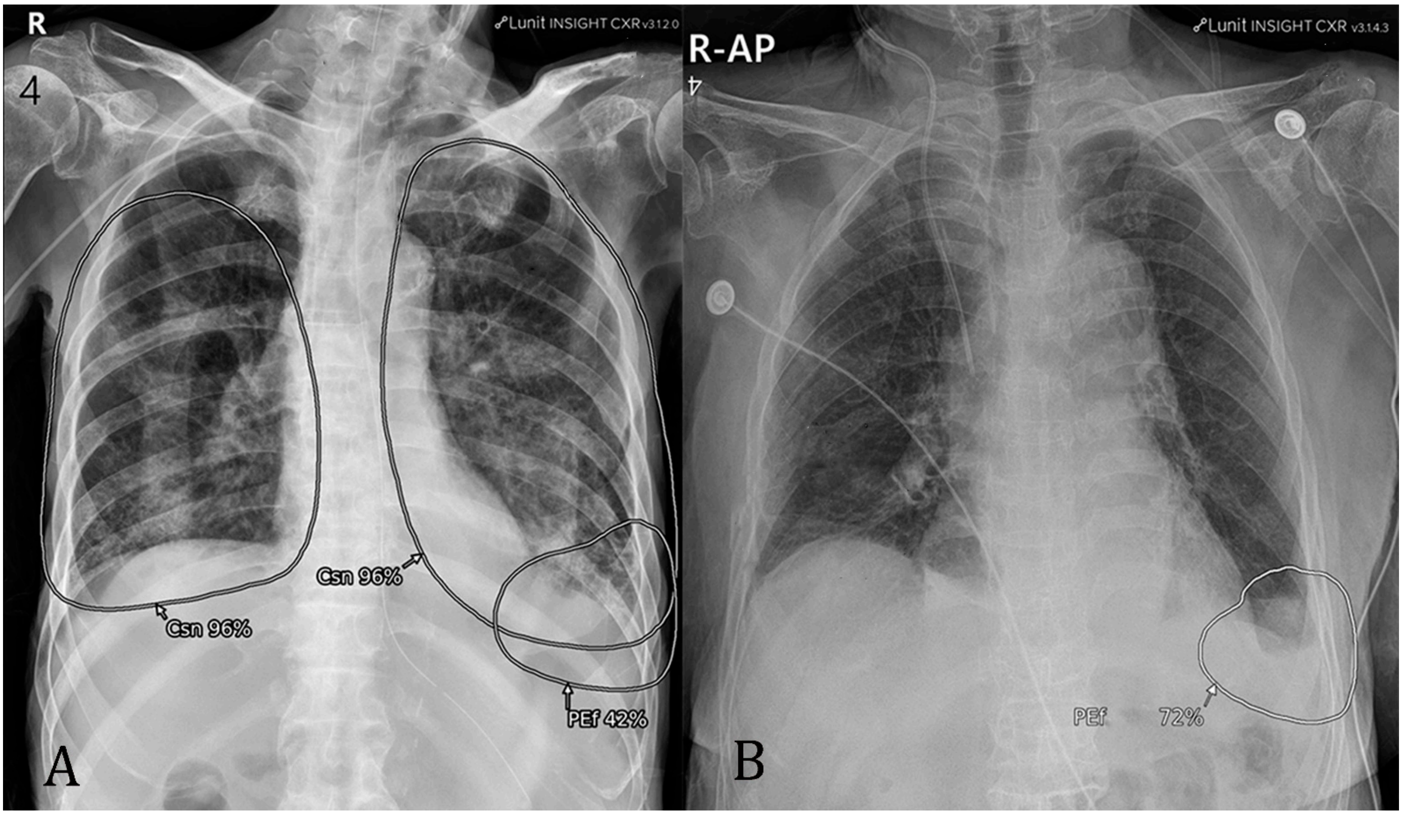

Figure S2. Differences in artificial intelligence-generated chest radiograph scores according to corticosteroid responsiveness and time category.

Abbreviations: CAT 0: category 0, 0-2 days from the onset of symptoms; CAT 1: category 1, 3-5 days from the onset of symptoms; CAT 2: category 3, 6-9 days from the onset of symptoms; CAT 3, more than 10 days from the onset of symptoms.

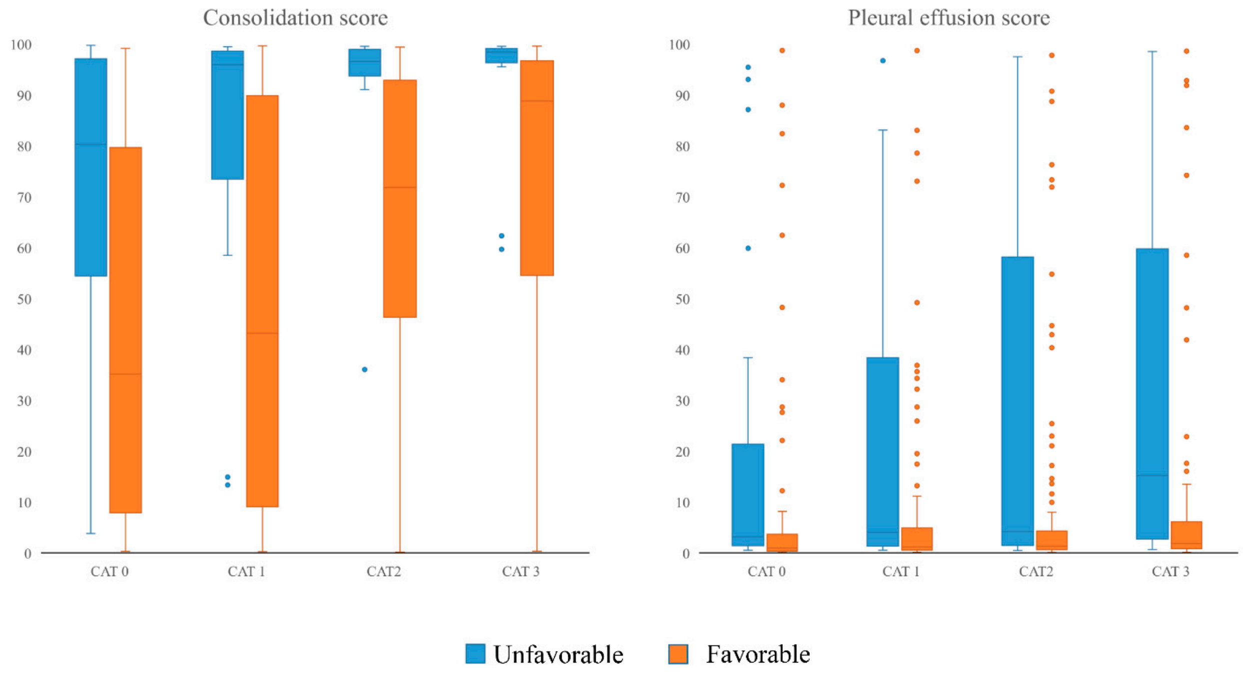

Figure S3. Association between consolidation score and unfavorable corticosteroid responsiveness according to the time category and subgroup.

Adjusted odds ratio and 95% confidence interval of consolidation score used for predicting corticosteroid responsiveness within each subgroup were calculated using multivariate logistic regression and adjusted for age, sex, Charlson comorbidity index, immune status, vaccination status, antiviral agent usage, and antibacterial agent usage.

Abbreviations: aOR, adjusted odds ratio; CI, confidence interval; CAT 0: category 0, 0-2 days from the onset of symptoms; CAT 1: category 1, 3-5 days from the onset of symptoms; CAT 2: category 3, 6-9 days from the onset of symptoms; CAT 3, more than 10 days from the onset of symptoms; CI, confidence interval.

		aOR (95% CI)	p-value
Total	After Jan, 2022	1.024 (0.999-1.050)	0.058
	Before Jan, 2022	1.020 (1.006-1.034)	0.004
	Non-hypoxia		
	Hypoxia	1.016 (1.004-1.029)	0.012
CAT 0	After Jan, 2022	1.029 (0.991-1.068)	0.136
	Before Jan, 2022	1.025 (0.995-1.057)	0.104
	Non-hypoxia		
	Hypoxia	1.036 (1.006-1.067)	0.019
CAT 1	After Jan, 2022	1.011 (0.981-1.041)	0.489
	Before Jan, 2022	1.043 (1.013-1.074)	0.005
	Non-hypoxia		
	Hypoxia	1.025 (1.004-1.046)	0.018
CAT 2	After Jan, 2022	1.031 (0.988-1.076)	0.161
	Before Jan, 2022	1.076 (1.011-1.145)	0.022
	Non-hypoxia		
	Hypoxia	1.054 (1.013-1.097)	0.010
CAT 3	After Jan, 2022	1.028 (0.972-1.087)	0.339
	Before Jan, 2022	1.036 (0.966-1.111)	0.322
	Non-hypoxia		
	Hypoxia	1.026 (0.974-1.081)	0.332

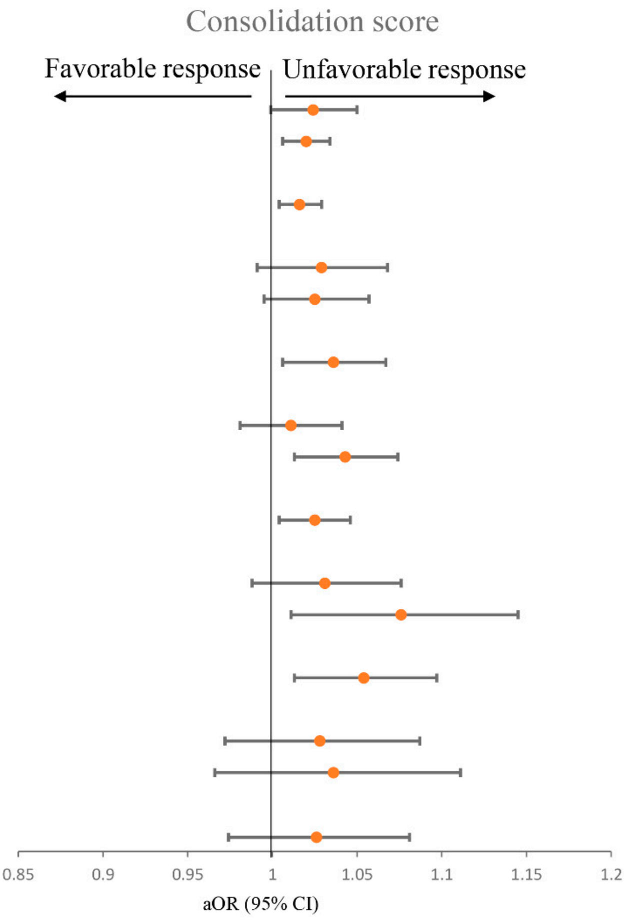

Figure S4. Association between pleural effusion score and unfavorable corticosteroid responsiveness according to the time category and subgroups.

[a] Adjusted odds ratio and 95% confidence interval of pleural effusion score used for predicting corticosteroid responsiveness within each subgroup were calculated using multivariate logistic regression and adjusted for age, sex, Charlson comorbidity index, immune status, vaccination status, antiviral agent usage, and antibacterial agent usage.

Abbreviations: aOR, adjusted odds ratio; CI, confidence interval; CAT 0: category 0, 0-2 days from the onset of symptoms; CAT 1: category 1, 3-5 days from the onset of symptoms; CAT 2: category 3, 6-9 days from the onset of symptoms; CAT 3, more than 10 days from the onset of symptoms; CI, confidence interval.

		aOR[a] (95% CI)	p-value
Total	After Jan, 2022	1.022 (1.005-1.039)	0.009
	Before Jan, 2022	1.006 (0.980-1.034)	0.652
	Non-hypoxia		
	Hypoxia	1.009 (0.995-1.022)	0.225
CAT 0	After Jan, 2022	1.072 (1.010-1.138)	0.021
	Before Jan, 2022	1.001 (0.941-1.066)	0.964
	Non-hypoxia		
	Hypoxia	1.005 (0.982-1.029)	0.663
CAT 1	After Jan, 2022	1.041 (1.005-1.078)	0.024
	Before Jan, 2022	1.007 (0.975-1.040)	0.677
	Non-hypoxia		
	Hypoxia	1.011 (0.993-1.030)	0.230
CAT 2	After Jan, 2022	1.035 (1.005-1.066)	0.032
	Before Jan, 2022	1.016 (0.979-1.055)	0.394
	Non-hypoxia		
	Hypoxia	1.015 (0.995-1.035)	0.134
CAT 3	After Jan, 2022	1.002 (0.973-1.032)	0.884
	Before Jan, 2022	1.010 (0.939-1.087)	0.787
	Non-hypoxia		
	Hypoxia	1.000 (0.975-1.026)	0.973

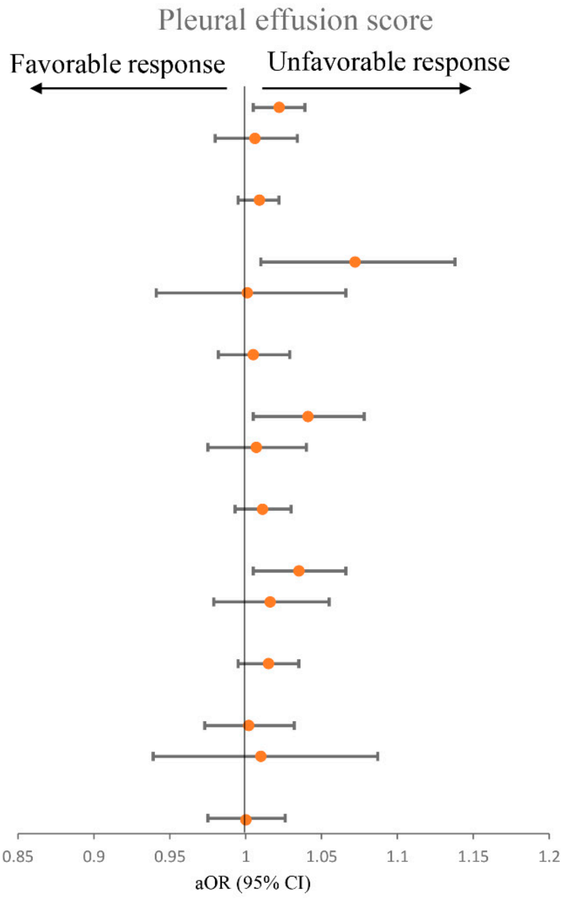

References

1. R&D Coronavirus disease (COVID-2019) R&D. Geneva: World Health Organization. Available online: http://www.who.int/blueprint/priority-diseases/key-action/novel-coronavirus/en/ (accessed August 3rd, 2020)
